# Heat shock transcription factor 1 facilitates liver cancer progression by driving super‐enhancer‐mediated transcription of MYCN

**DOI:** 10.1002/cam4.70157

**Published:** 2024-09-09

**Authors:** Yizhe Liu, Qili Shi, Yue Su, Zhiao Chen, Xianghuo He

**Affiliations:** ^1^ Fudan University Shanghai Cancer Center and Institutes of Biomedical Sciences; Department of Oncology Shanghai Medical College, Fudan University Shanghai China; ^2^ Key Laboratory of Breast Cancer in Shanghai, Fudan University Shanghai Cancer Center Fudan University Shanghai China; ^3^ Shanghai Key Laboratory of Radiation Oncology, Fudan University Shanghai Cancer Center Fudan University Shanghai China

**Keywords:** HSF1, liver cancer, MYCN, super‐enhancer, transcriptional regulation

## Abstract

**Background:**

Heat shock transcription factors (HSFs) play crucial roles in the development of malignancies. However, the specific roles of HSFs in hepatocellular carcinoma (HCC) have yet to be fully elucidated.Aims:To explore the involvement of the HSF family, particularly HSF1, in the progression and prognosis of HCC.

**Materials & Methods:**

We conducted a thorough analysis of HSF expression and copy number variations across various cancer datasets. Specifically focusing on HSF1, we examined its expression levels and prognostic implications in HCC. In vitro and in vivo experiments were carried out to evaluate the impact of HSF1 on liver cancer cell proliferation. Additionally, we utilized CUT&Tag, H3K27 acetylation enrichment, and RNA sequencing (RNA‐seq) to investigate the super‐enhancer (SE) regulatory landscapes of HSF1 in liver cancer cell lines.

**Results:**

HSF1 expression is elevated in HCC and is linked to poor prognosis in several datasets. HSF1 stimulates liver cancer cell proliferation both in vitro and in vivo, partly through modulation of H3K27ac levels, influencing enhancer distribution. Mechanistically, our findings demonstrate that HSF1 transcriptionally activates MYCN expression by binding to its promoter and SE elements, thereby promoting liver cancer cell proliferation. Moreover, increased MYCN expression was detected in HCC tumors and correlated with unfavorable patient outcomes.

**Discussion:**

Our study sheds light on previously unexplored aspects of HSF1 biology, identifying it as a transcription factor capable of shaping the epigenetic landscape in the context of HCC. Given HSF1's potential as an epigenetic regulator, targeting the HSF1‐MYCN axis could open up new therapeutic possibilities for HCC treatment.

**Conclusion:**

The HSF1‐MYCN axis constitutes a transcription‐dependent regulatory mechanism that may function as both a prognostic indicator and a promising therapeutic target in liver cancer. Further exploration of this axis could yield valuable insights into novel treatment strategies for HCC.

## Graphical abstract

In the present study, we presented the HSF1‐MYCN regulatory axis, a transcription‐dependent regulatory axis, to reveal the molecular mechanism of Hepatocellular Carcinoma (HCC) proliferation. HSF1 alters the overall H3K27ac levels and super‐enhancer (SE) distribution in HCC cells.

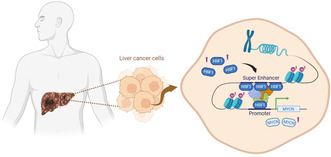

## 1 INTRODUCTION

Hepatocellular carcinoma (HCC) is a highly aggressive primary liver malignancy that is the second leading cause of cancer‐related death, and its incidence is increasing worldwide.^1^, ^2^, ^3^ Medical technologies, such as surgical resection, chemotherapy, radiation therapy, and the emerging modality of immunotherapy, have advanced over the past decades.^4^, ^5^, ^6^ However, success in improving the survival rates of HCC patients remains limited. Therefore, it is crucial to identify and characterize oncogenes associated with HCC, to study the related molecular mechanisms in depth, and to develop clinical therapeutic applications.

Eukaryotic cells depend on a tightly regulated state of protein homeostasis to perform their functions. Under stressful conditions, the heat shock response (HSR), a sophisticated mechanism, is activated to ensure cell survival.^7^, ^8^ Heat shock transcription factors (HSFs) can bind to the promoter and enhancer regions of genes encoding heat shock proteins (HSPs) and markedly prime their transcription. Then, transcriptionally generated HSPs act to prevent the aggregation of misfolded and malfunctioning proteins as effectors of the HSR to minimize the toxic effects of these abnormal proteins.^9^, ^10^, ^11^ In Homo sapiens, the major HSPs include HSF1, HSF2, HSF4, HSFY, and HSFX. However, only HSF1, HSF2, and HSF4 have the capability to bind to genomic DNA. Specifically, HSF2 is involved in corticogenesis and spermatogenesis, and HSF4 is required for the maintenance of sensory organs, such as the lens and olfactory epithelium.^12^, ^13^ However, HSF1 acts as a major regulator of the HSR; it can be expressed in all human tissues and responds rapidly and robustly to protein toxicity.^14^

Furthermore, HSF1 exerts influence on the transcription of a wide array of genes. In cultured cancer cells, HSF1 regulates genes associated with proliferation, protein synthesis, and glucose metabolism, thereby facilitating malignant transformation and impacting various cellular functions.^15^, ^16^, ^17^ Interestingly, HSF1 could promote tumorigenesis in cancer cells without HSR, and multiple target genes of HSF1 were identified in cancer cells but not in heat‐shock‐treated cells.^18^ In HCC, the mammalian target of rapamycin (mTOR) has been shown to phosphorylate HSF1 at serine 326, which is conductive to the activation of HSF1.^19^ Jin et al. proposed a pathogenic mechanism whereby HSF1 activation promoted growth of premalignant cells and HCC development by stimulating lipid biosynthesis and perpetuating chronic hepatic metabolic disease induced by carcinogens.^20^ Furthermore, Li et al. observed that HSF1 directly activated miR‐135b expression and enhanced HCC cell motility and invasiveness through the HSF1/miR‐135b/RECK&EVI5 axis.^21^ However, the specific molecular mechanism of how HSF1 activates oncogenes needs to be elucidated.

Transcriptional regulatory elements in complex genomes are key factors in the dynamic regulation of the genome during cell development, in response to external stimuli and under disease conditions. Alterations in these elements are closely related to cancer, where genetic changes and dysregulation of gene expression programs lead to a change from a normal to a cancerous cell state.^22^, ^23^ Transcription factors (TFs) have the ability to bind to promoters and enhancers near the loci of oncogenes, thereby regulating their transcription, and the binding of TFs is often accompanied by the presence of accessible chromatin and abundant histone modifications near the corresponding DNA.^24^ Histone H3 lysine 27 acetylation (H3K27ac) and H3 lysine 4 trimethylation (H3K4me3) marks are commonly used to identify promoter regions, and H3K27ac and H3 lysine 4 monomethylation (H3K4me1) marks are commonly used to identify enhancer regions. Additionally, high levels of H3K27ac and enriched mediators can be labeled as markers to identify super‐enhancer (SE) regions.^25^ SEs are broader clusters of enhancers with a median size of 19.4 kb and are usually distinguished from typical enhancers by their size and mediator level.^26^ Histone modifications in these regions recruit TFs and other protein complexes, such as transcription initiation complexes, to facilitate gene transcription. Conversely, studies have also shown that the binding of TFs can influence changes in the abundance of histone modifications, and the temporal order of these events is not yet clearly established.^27^, ^28^, ^29^ So far, the effect of HSF1 on SEs and related histone modifications has not been reported in HCC.

In this study, we analyzed the HSF family and identified HSF1, which had the most significant copy number variation (CNV) and was most strongly associated with poor patient prognosis, as the research focus. HSF1 facilitated the proliferation of HCC cells in vitro and in vivo, and affected the H3K27ac abundance and the distribution of SE in Huh7 cells. By combining the results of several next‐generation sequencing analyses, we found that HSF1 binds to the promoter and SE regions of the oncogene MYCN and changes its histone modifications, thereby regulating its transcription. MYCN also affected the proliferation of HCC cells, and patients with higher MYCN expression had poorer clinical prognoses. In addition, we demonstrated that HSF1 promoted HCC cell proliferation through MYCN. In conclusion, our study suggests that the HSF1–MYCN axis is a potentially robust biomarker and therapeutic target for HCC.

## 2 MATERIALS AND METHODS

### 2.1 CCK‐8 and colony formation assays

First, liver cancer cells were seeded into 96‐well plates (1500–3000 cells/well) in quadruplicate. Cell proliferation was measured on Days 1, 3, 5, and 7 using CCK‐8 reagent. For colony formation assay, cells were seeded into six‐well plates (1500–3000 cells/well) and cultured for weeks. The resulting colonies were calculated after staining with 1.25% crystal violet.

### 2.2 In vivo tumorigenesis assay

For each group, a total of 3 × 10^6^ cells were injected subcutaneously into the right dorsal side of each recipient BALB/c‐nu/nu mice (6 weeks old, female). The general behavior of the mice was monitored, and tumor volumes (*V* = (length × width^2^)/2) were measured every 4 days for 35 days. These mice were humanely sacrificed 35 days after injection, and subcutaneous tumors were harvested and weighed.

### 2.3 ChIP‐seq

First, liver cancer cells were treated with 1% formaldehyde for 10 min for crosslinking, and 0.125 M glycine was then used to terminate the crosslinking reaction. After the cells were washed and collected, chromatin was digested with 0.5 μL of micrococcal nuclease to cleave DNA into fragments. SimpleChIP Chromatin IP buffers (Cell Signaling Technology) were used to extract and resuspend the protein–DNA complexes.

The steps for immunoprecipitation were similar to those for coimmunoprecipitation (Co‐IP). For qRT‐PCR analysis or DNA sequencing (DNA‐seq), the target DNA was washed and eluted with MinElute Spin Columns (Qiagen). To construct libraries, a DNA‐seq kit from NEB was used following the manufacturer's instructions. The final library was sorted using DNA magnetic beads (Vazyme) to obtain a ChIP‐seq library containing fragments of 250–500 bp. DNA samples were subjected to paired‐end, 100 × 100 cycle sequencing on an Illumina HiSeq 2000 instrument. To verify the ChIP‐seq results, ChIP–qPCR was performed. A list of the primers used for qRT‐PCR is provided in Table S1.

The raw sequencing data were mapped to the hg38 build of the human genome using Bowtie2. DeepTools was used to convert Bam files into BigWig files. Peaks were called using the MACS2 peak caller and were then annotated with Homer. The distributions of the peaks of interest along the genomic regions of genes were visualized with IGV.

### 2.4 CUT&Tag

Huh7 cells incubated at 37°C were harvested, counted, split into groups of 100,000 cells each, and centrifuged for 3 min at 500*g* at 4°C. The CUT&Tag experiment was then performed according to the manufacturer's protocol (Novoprotein), using an HSF1 rabbit antibody (Proteintech) as the primary antibody and a goat anti‐rabbit IgG (Abcam) as the secondary antibody. A negative control group was included without the primary antibody, and a positive control group was included with H3K27ac (Active Motif) as the primary antibody. The resulting sequencing data were processed, analyzed, and visualized using the same methods as ChIP‐seq, employing software including Bowtie2, DeepTools, MACS2, and IGV.

### 2.5 RNA‐seq analysis

In brief, total RNA of Huh7 cells was extracted using TRIzol reagent. RNA libraries were constructed by poly(A) selection. The sequencing library was prepared using an Illumina mRNA‐seq sample preparation kit and was then subjected to paired‐end sequencing on the Illumina HiSeq platform. Raw sequencing reads from RNA‐seq were first processed to remove adapter sequences using Cutadapt (version 3.2). After trimming, the cleaned paired‐end reads were aligned to the human reference genome GRCh38 using the HISAT2 (version 2.2.1)^30^ aligner. Read counts were quantified by featureCounts (version 2.0.1)^31^ using GENCODE V35 and normalized to TPM. Genes with a fold change greater than 1.5 or less than 2/3 were considered differentially expressed. Using the R package clusterProfiler, we performed a Gene Ontology overrepresentation analysis to identify biological processes for genes downregulated (fold change <2/3) by siHSF1 and siMYCN, as determined from RNA‐seq data.

### 2.6 Super‐enhancer analysis

Rank ordering of super‐enhancers (ROSE) algorithm was used to define SEs.^26^, ^32^ The distance of stitching enhancers was fixed at 12.5 kb. Default Settings were used for all other parameters. SE‐related genes were identified by the ROSE_geneMapper.py program and obtained from the ROSE EHANCER_TO_TOP_GENE.txt file.

### 2.7 Data collection

RNA‐seq and clinical data of HCC and 21 other cancer types were downloaded from TCGA (https://portal.gdc.cancer.gov), the International Cancer Genome Consortium (ICGC; https://dcc.icgc.org/projects/LIRI‐JP), the National Omics Data Encyclopedia (NODE; https://www.biosino.org/node/project/detail/OEP000321) and the NCBI Gene Expression Omnibus (GEO) under accession numbers GSE77314 and GSE144269. (version 2.16.3). The CNV data were obtained from cBioPortal. All RNA‐seq data were transformed into TPM for downstream analysis. Differentially expressed genes (DEGs) between tumors and adjacent normal tissues were identified using the edgeR package with count data. Multiple testing corrections were performed using the Benjamini‐Hochberg method to control the False Discovery Rate (FDR). Fold change refers to the ratio of gene expression levels between tumors and adjacent normal tissues.

### 2.8 Statistical analysis

At least three independent replicates were performed for each experiment, and the results are expressed as the mean ± SEM. Student *t*‐tests (two‐tailed) and one‐way ANOVA were used to compare the means of two or more samples unless otherwise stated. We used the Kaplan–Meier method to calculate survival curves and a log‐rank test to check whether gene levels were significantly associated with overall patient survival. A *p* < 0.05 was considered significant. All statistical analyses were performed using GraphPad Prism 7 or R. Details of the statistical analysis are indicated in the figure legends.

## 3 RESULTS

### 3.1 High expression of HSF1 correlates with poor outcomes in patients with HCC

**FIGURE 1:**
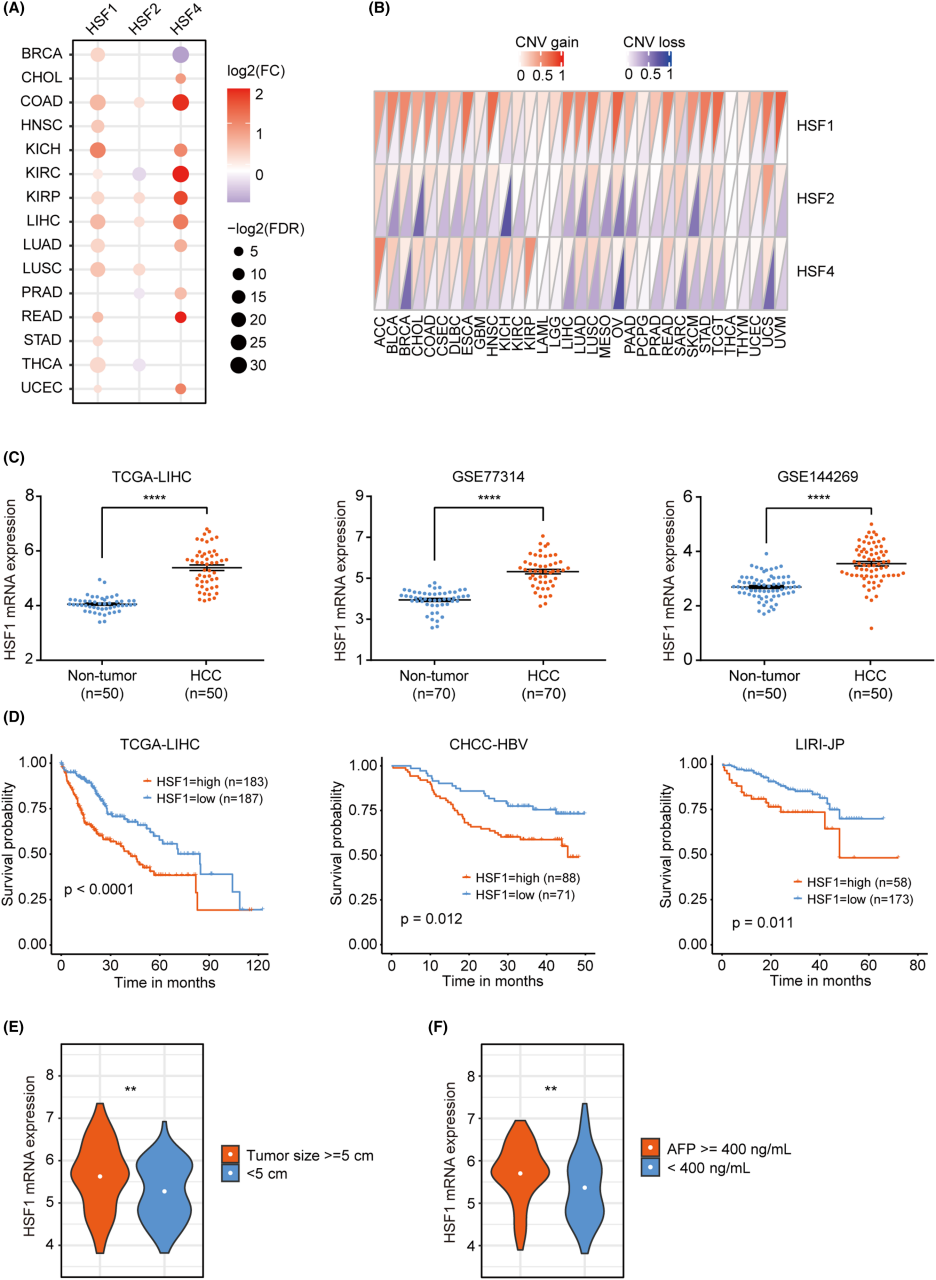
High expression of HSF1 correlates with poor outcomes in patients with HCC. (A) Differential expression analysis of the HSF family members HSF1, HSF2, and HSF4 in 15 cancer types. (B) Copy number variation analysis of HSF1, HSF2, and HSF4 in 33 cancer types. (C) Analysis of HSF1 expression in tumor and non‐tumor tissues from three groups of HCC patients. (D) The relationship between high and low expression of HSF1 and prognosis in three separate groups of HCC patients. (E, F) Clinical significance of HSF1 in patients with HCC; high HSF1 expression was positively correlated with tumor size (> = 5 cm) and AFP level (> = 400 ng/mL). The values are expressed as the means ± SEMs (C, E, F). **p < 0.01; ****p < 0.0001 by one‐way ANOVA or two‐tailed Student's *t*‐test.

Among the members of the HSF family, HSF1, HSF2, and HSF4 are widely expressed in various cancers and tissues. We therefore investigated the perturbations in the expression of these three genes across 22 cancer types with paired adjacent non‐tumor control tissues. At least one HSF gene was differentially expressed in 15 of these cancer types (Figure 1A). Importantly, HSF1 and HSF4 showed significantly higher expression in most cancer types. In particular, all three genes were upregulated in colon adenocarcinoma (COAD), liver hepatocellular carcinoma (LIHC), and kidney renal papillary cell carcinoma (KIRP). To explore whether genetic alterations can affect their expression levels, we further analyzed CNVs in these three genes across 33 cancer types. Intriguingly, HSF1 showed extensive copy number amplification in all cancer types (Figure 1B). In addition, in The Cancer Genome Atlas (TCGA)‐LIHC cohort, samples with HSF1 copy number amplification had higher HSF1 expression than those without amplification (Figure S1A).

We next targeted HSF1 independently and analyzed its expression in different subsets of liver cancer patients, along with the relationship between its expression and patient prognosis. Surprisingly, we found the same pattern in three different sets of HCC patients. In comparison to non‐tumor tissues, the mRNA expression level of HSF1 was notably increased in HCC tissues (Figure 1C). Furthermore, analysis of CPTAC sample data from UALCAN^33^, ^34^ demonstrated significantly elevated protein levels of HSF1 in primary tumor tissues compared to normal tissues (Figure S1B). Additionally, a high level of HSF1 was significantly associated with a low survival probability among liver cancer patients (Figure 1D). In addition, the expression of HSF1 was positively correlated with the tumor size and alpha‐fetoprotein (AFP) level (Figure 1E,F).

### 3.2 High expression of HSF1 promotes liver cancer cell proliferation in vitro and in vivo

**FIGURE 2:**
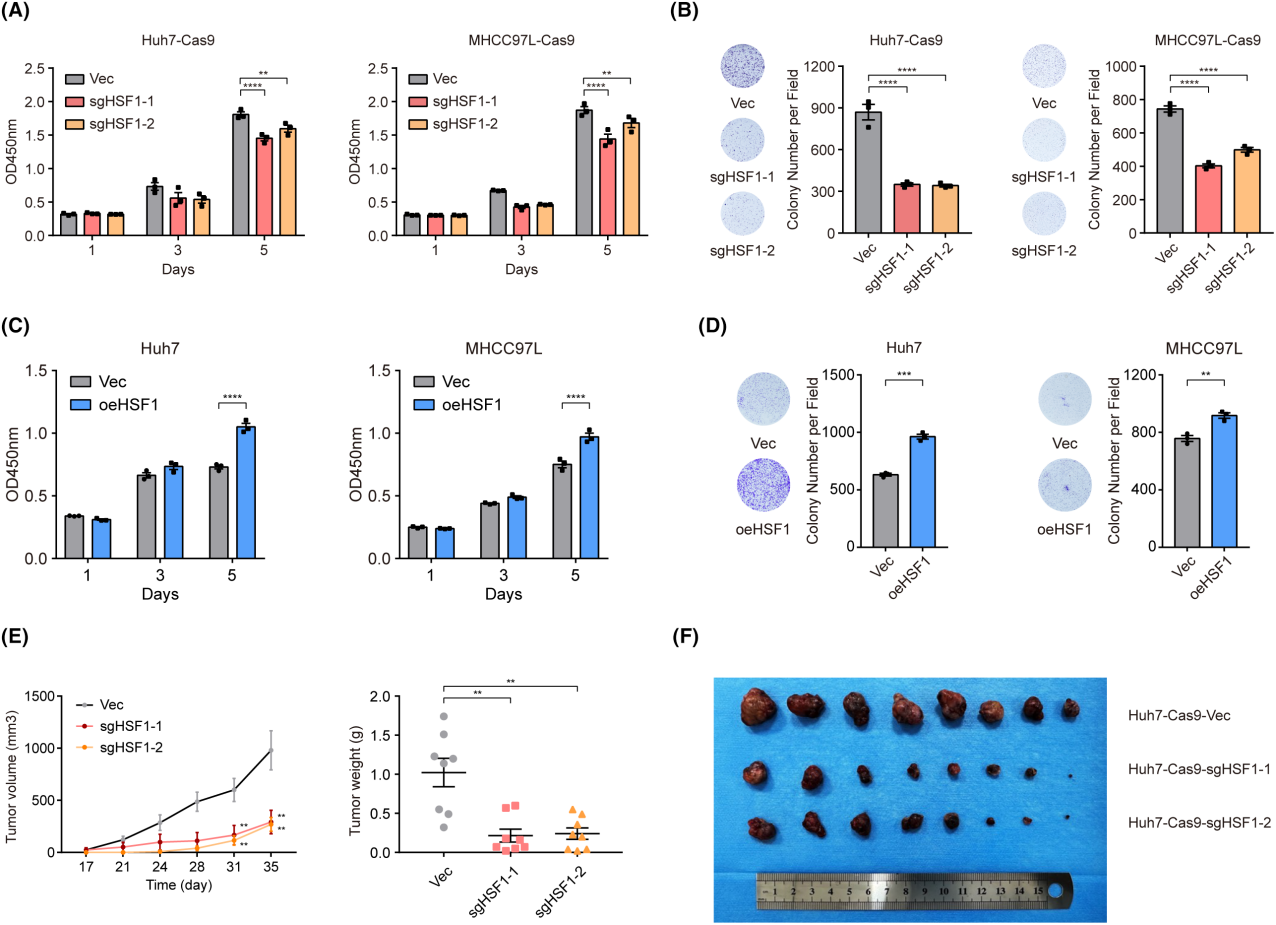
High expression of HSF1 promotes liver cancer cell proliferation in vitro and in vivo. (A, B) CCK‐8 assay and colony formation assay of the two liver cancer cell lines with HSF1 knockout (*n* = 3). (C, D) CCK‐8 assay and colony formation assay of the Huh7 and MHCC97L cells after HSF1 overexpression (*n* = 3). (E) Tumor volumes and weights were assessed in both the sgHSF1 and negative control groups of xenograft mice (*n* = 8). (F) Images of xenografts from nude mice carrying subcutaneous xenografts derived from sgHSF1 cells or control cells are presented. The values are expressed as the means ± SEMs (A–E). **p < 0.01; ***p < 0.001; ****p < 0.0001 by one‐way ANOVA or two‐tailed Student's *t*‐test.

To determine the biological function of HSF1 in liver cancer cells, we first used CRISPR/Cas9 technology to suppress endogenous HSF1 expression in Huh7 and MHCC97L cells (Figure S1C). The CCK‐8 assay and colony formation assay were used to evaluate the proliferation ability of liver cancer cells. Based on the absorbance at 450 nm on the last day, liver cancer cell proliferation was significantly impaired after knockout of HSF1 (Figure 2A). The photographs of cell plates and clonal statistics revealed that the colony‐forming capability was reduced in the sgRNA treatment groups of both Huh7‐Cas9 and MHCC97L‐Cas9 cells (Figure 2B). Similarly, we knocked down endogenous HSF1 expression with siRNAs in Huh7 and MHCC97L cells. The mRNA and protein levels of HSF1 were significantly decreased in both cell lines (Figure S1D). After knockdown of HSF1, the proliferation and colony formation ability of HCC cells were also significantly decreased (Figure S1E).

To further elucidate the function of HSF1, we overexpressed HSF1 in Huh7 and MHCC97L cells (Figure S1F). Compared to the control group, HSF1 overexpression significantly enhanced the proliferation and colony formation ability of both HCC cell lines (Figure 2C,D). Moreover, the impact of HSF1 on the proliferation of liver cancer cells was evaluated in vivo. Stably HSF1‐knockout cells, derived from Huh7‐Cas9, were subcutaneously transplanted into nude mice. Notably, tumor volumes and weights in the FBL knockout group exhibited a significant decrease compared to the control group (Figure 2E,F). Taken together, these results strongly indicate that HSF1 plays a pivotal role in enhancing the proliferation of liver cancer cells, both in vitro and in vivo.

### 3.3 HSF1 affects the super‐enhancer variation in liver cancer cells

**FIGURE 3:**
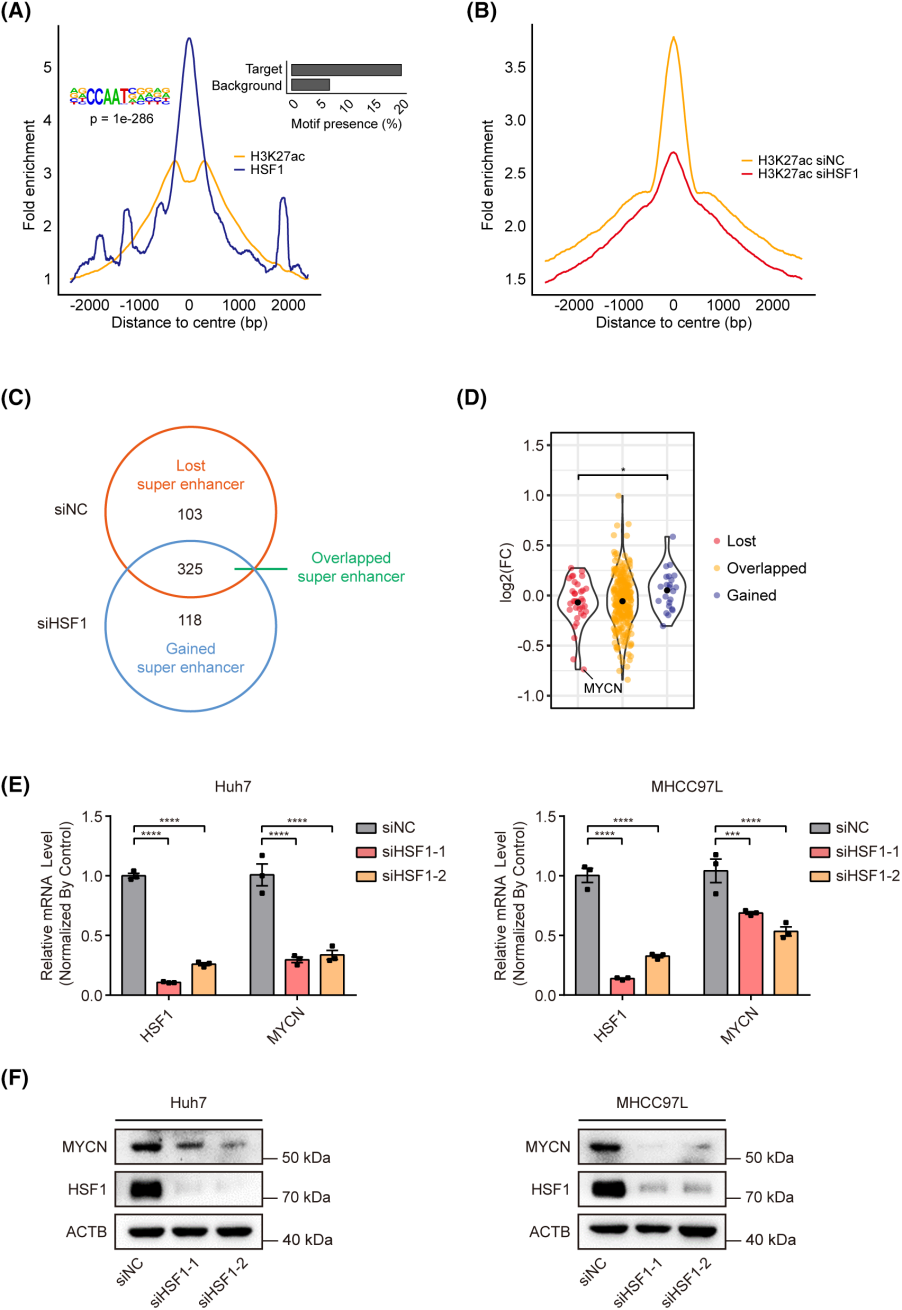
HSF1 affects the super‐enhancer (SE) variation in liver cancer cells. (A) Line plots showing CUT&Tag signals of HSF1 and ChIP‐seq signals of H3K27ac at the center of peaks in Huh7 cells. (B) Line plots showing ChIP‐seq signals of H3K27ac at the center of peaks in Huh7 cells transfected with siNC or siHSF1. (C) Venn diagram showing the lost SEs (only in the siNC group), overlapped SEs (in both the siNC and siHSF1 groups), and gained SEs (only in the siHSF1 group) and the number in each group. (D) Violin plot showing the changes in the mRNA levels of genes in the lost, overlapped, and gained SE groups. (E, F) Relative mRNA and protein expression levels of MYCN upon knock‐down of HSF1 in Huh7 and MHCC97L cells (*n* = 3). The values are expressed as the means ± SEMs (D, E). *p < 0.05; ***p < 0.001; ****p < 0.0001 by one‐way ANOVA or two‐tailed Student's *t*‐test.

Previous studies showed that TFs may affect epigenetic changes, including histone modifications.^28^ To explore the potential broader epigenetic effects of HSF1 beyond its classical role as a transcription factor in gene expression regulation, we employed three essential experimental approaches in Huh7 cells. Firstly, we performed CUT&Tag using HSF1 as the primary antibody. We analyzed the peaks and motifs in the HSF1 CUT&Tag and H3K27ac ChIP‐seq data in Huh7‐null cells, and we found that they exhibited a high degree of overlap near the peak center and were similarly enriched at the approximate motif CCAAT (Figure 3A). This pattern suggests a potential regulatory relationship where HSF1 influences H3K27ac levels.

Next, we employed ChIP‐seq with an H3K27ac antibody both before and after HSF1 knockdown. Upon HSF1 knockdown, a decrease in the overall peak heights of H3K27ac was observed (Figure 3B), indicating that HSF1 influences the global activity of H3K27ac in Huh7 cells. To investigate whether these changes in histone modifications reflect alterations in enhancer activity, we used the ROSE software to identify SE activity before and after HSF1 knockdown. The results revealed that upon HSF1 knockdown, some SEs were lost, while some SEs that were silenced in the control group became activated (Figure 3C).

Lastly, RNA‐seq was performed before and after HSF1 knockdown to analyze changes in the transcriptomic. By predicting the SE‐regulated nearby genes and integrating the results of RNA‐seq analysis, we further found an overall decrease in mRNA levels in the subset of genes with lost SEs and an increase in the mRNA levels of genes in the group with newly activated SEs. Furthermore, the mRNA levels differed significantly between these two groups of genes (Figure 3D). In summary, our findings indicate that HSF1 functions not only as a transcription factor regulating gene transcription in liver cancer cells but also induces global changes in H3K27ac and alters the distribution of super enhancers. HSF1 may further modulate gene expression by affecting the activity of SEs.

To identify representative genes regulated by HSF1 through SEs, our analysis revealed MYCN as the gene displaying the most significant change in mRNA levels among the group of lost SEs. To determine whether MYCN expression is regulated by HSF1, the siRNAs of HSF1 were used to knock down HSF1 expression both in Huh7 and MHCC97L cells. The mRNA expression variation was detected by qRT‐PCR (Figure 3E), and the protein level changes were detected by Western blot (Figure 3F). The results indicated that in conjunction with the downregulation of HSF1, MYCN expression was also downregulated, suggesting that MYCN is a downstream target of HSF1 in liver cancer cells.

### 3.4 HSF1 transcriptionally activates MYCN expression via occupation of its promoter and super‐enhancer element

**FIGURE 4:**
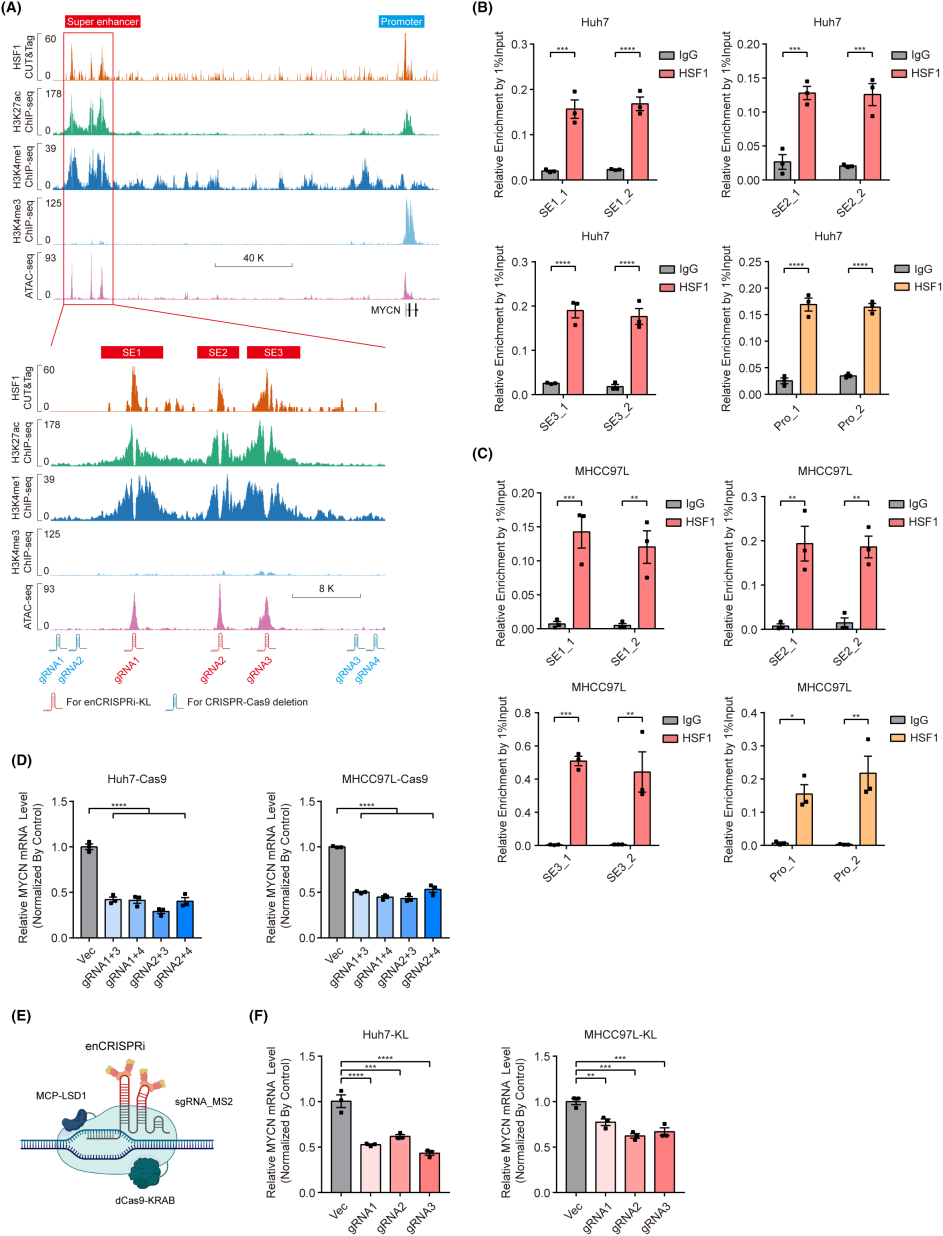
HSF1 transcriptionally activates MYCN expression via occupation of its promoter and SE element. (A) Profiles of HSF1, H3K27ac, H3K4me1, and H3K4me3 occupancy and ATAC‐seq peaks at the MYCN promoter (blue rectangle) and SE (red rectangle) regions in Huh7 cells. The red box indicates the occupancy of HSF1, which contains three specific constituent SEs (SE1, SE2, and SE3). The sgRNAs were esigned on the basis of the abovementioned peaks. (B, C) ChIP–qPCR analysis of HSF1 enrichment in the promoter and SE regions of MYCN in Huh7 and MHCC97L cells (*n* = 3). (D) Relative MYCN mRNA levels upon knockout of the SE regions by CRISPR–Cas9 gene editing (*n* = 3). (E) Schematic diagram of the enCRISPRi system containing the dCas9‐KRAB fusion protein, the MCP‐LSD1 fusion protein, and a sgRNA with two MS2 hairpins. (F) Relative MYCN mRNA levels upon blocking the SE with three individual sgRNAs in Huh7‐KL and MHCC97L‐KL cells (*n* = 3). The values are expressed as the means ± SEMs (B–D, F). *p < 0.05; **p < 0.01; ***p < 0.001; ****p < 0.0001 by one‐way ANOVA or two‐tailed Student's *t*‐test.

To explore how HSF1 regulates MYCN expression in liver cancer cells, we further investigated the regulatory mechanism of HSF1 on MYCN at the transcriptional level. ChIP‐seq of H3K4me3 and H3K4me1 was complemented in Huh7‐null cells to accurately identify promoter and enhancer elements on the genome. Moreover, the assay for transposase accessible chromatin using sequencing (ATAC‐seq) was used to locate open regions in chromatin which may contain transcriptional activity. Combined, the H3K4me1, H3K27ac, and ATAC‐seq signal defined the presence of a SE of approximately 20 kb in length located 150 kb upstream of the MYCN locus (Figure 4A). This large area of the SE contained three peaks of ATAC‐seq and three peaks of HSF1 CUT&Tag, suggesting that HSF1 may bind to all three SE regions. Notably, prominent enrichment of H3K27ac signaling near the CUT&Tag peak of HSF1 was observed, suggesting a direct correlation between the SE‐mediated transcriptional activation of HSF1 and its binding to the corresponding DNA region. In addition, analysis of H3K27ac, H3K4me3, and H3K4me1 ChIP‐seq data and HSF1 CUT&Tag data generated from Huh7 cells, we found that both the promoter and SE regions of MYCN are occupied by HSF1. We designed two separate pairs of primers targeting each of the three SE regions and promoter region to confirm the abovementioned results by ChIP–quantitative PCR (ChIP–qPCR) (Figure 4B,C). The results indicated that HSF1 functions as a transcription factor and directly binds to the promoter and SE of MYCN in liver cancer cells.

To explore whether the SE upstream of MYCN can actually regulate MYCN mRNA expression, we used two methods for verification. Initially, we designed a set of single‐guide RNAs (sgRNAs) spanning the SE region and deleted the entire MYCN SE by CRISPR–Cas9 gene editing. We used different pairs of the two sgRNAs targeting the region upstream of the SE and the two sgRNAs targeting the region downstream and found that the mRNA level of MYCN was significantly decreased when the MYCN SE region was deleted with all four sgRNA combinations (Figure 4D). Genomic DNA was extracted from the corresponding cells, and PCR was performed using them as templates to verify the knockout efficiency of the SE. We used an extension time of 1 min to ensure that there would be no amplification of DNA fragments in the control group, while the templates of the knockout SE group were able to amplify short fragments of DNA from both ends (Figure S2A). Next, we used the enhancer‐targeting CRISPR interference (enCRISPRi) system designed and constructed by Li et al.^35^ to infect Huh7 and MHCC97L cells, fuse dCas9 to the KRAB protein, and recruit the lysine‐specific demethylase MCP‐LSD1 with MS2‐sgRNA (Figure 4E). With an sgRNA designed to target the central region of the SE, the activity of the SE in the corresponding region can be inhibited. Through qRT‐PCR, we found that three independent sgRNAs exerted their effects by guiding the enCRISPRi system, thus inhibiting the mRNA expression of MYCN (Figure 4F). In conclusion, these results demonstrated that HSF1 occupies both the promoter and SE regions of MYCN, thus activating its transcription in liver cancer cells.

### 3.5 HSF1 promoted the proliferation of liver cancer cells by regulating MYCN

**FIGURE 5:**
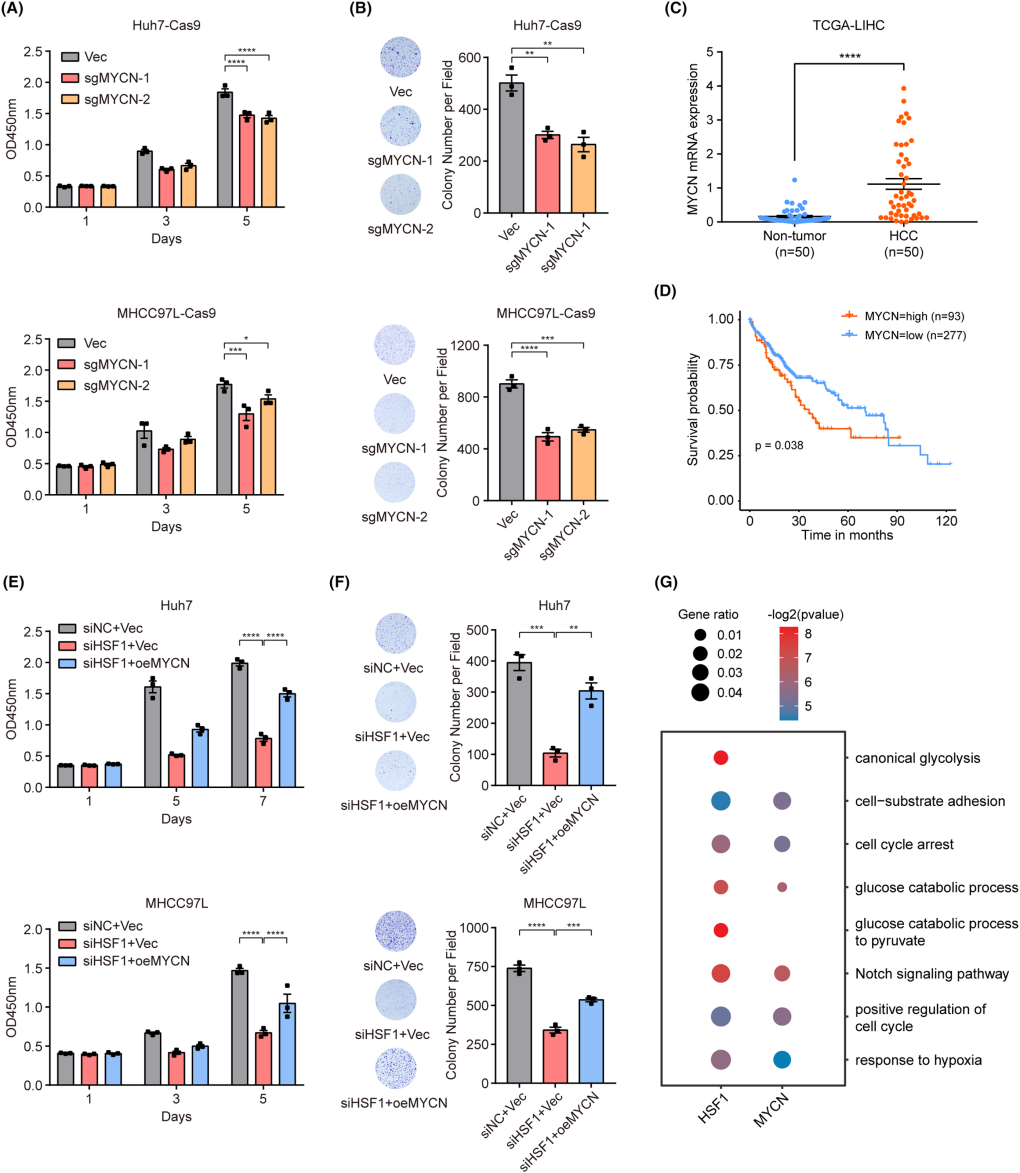
HSF1 promoted the proliferation of liver cancer cells by regulating MYCN. (A) CCK‐8 assay of Huh7‐Cas9 and MHCC97L‐Cas9 cells infected with two independent lentiviruses containing different sgRNAs (*n* = 3). (B) Colony formation assay of Huh7‐Cas9 and MHCC97L‐Cas9 cells in the NC and MYCN knockout groups (*n* = 3). (C) Analysis of MYCN expression in tumor and non‐tumor tissues from HCC patients. (D) Relationships between high and low expression of MYCN and the prognosis of HCC patients. (E) Viability of Huh7 and MHCC97L cells treated as indicated. (F) Colony formation assays and colony counts of Huh7 and MHCC97L cells treated as indicated. The values are expressed as the means ± SEMs (A–C, E, F). (G) Gene Ontology Biological Process (GO‐BP) overrepresentation analysis of genes downregulated (fold change <2/3) by siHSF1 and siMYCN, identified via RNA‐seq. The size of each dot represents the gene ratio. The color of each dot indicates the *p*‐value (−log2). *p < 0.05; **p < 0.01; ***p < 0.001; ****p < 0.0001 by one‐way ANOVA or two‐tailed Student's *t*‐test.

To investigate the effect of MYCN expression on the growth of liver cancer cell lines, we designed sgRNAs targeting the exons of MYCN, and verified the function of MYCN in the Huh7‐Cas9 and MHCC97L‐Cas9 cell lines. The mRNA and protein level of MYCN was significantly decreased in both liver cancer cell lines after infection with lentiviruses containing the sgRNAs (Figure S2B). The absorbance at 450 nm in the sgMYCN groups was visibly lower than that in the control groups on Day 5, revealing that knockout of MYCN exerted a significant inhibitory effect on the proliferative capacity of liver cancer cells (Figure 5A). Additionally, knockdown of MYCN reduced the colony forming ability of liver cancer cell lines. Fewer and smaller colonies were formed in the experimental group than in the control group (Figure 5B). Furthermore, the mRNA level of MYCN was observably higher in HCC tissues than in non‐tumor tissues, and a high MYCN level in HCC patients was clearly associated with a low survival probability (Figure 5C,D).

In order to investigate whether HSF1 promotes the proliferation of liver cancer cells by regulating MYCN, we designed and conducted a rescue experiment in Huh7 and MHCC97L cell lines. After knockdown of HSF1 with siRNAs, MYCN expression was restored by lentivirus infection. The mRNA and protein expression of HSF1 and MYCN were detected both in Huh7 and MHCC97L cell lines. MYCN mRNA and protein levels were decreased after knockdown of HSF1 and recovered after lentivirus infection (Figure S2C). Then, CCK‐8 assay and colony formation assay were used to examine the proliferation ability of different groups of cells. The results revealed that restoring MYCN expression in the case of HSF1 knockdown could significantly restore cell proliferation both in Huh7 and MHCC97L cells (Figure 5E,F). Taken together these results indicated that HSF1 promoted the proliferation of liver cancer cells by regulating MYCN.

To investigate the pathway through which HSF1 regulates MYCN to promote the proliferation of HCC cells, we performed transcriptome sequencing after MYCN knockdown in Huh7 cells. Additionally, we integrated the transcriptome sequencing data from HSF1 knockdown experiments and conducted GO enrichment analysis on the co‐differentially expressed genes of HSF1 and MYCN knockdown. This analysis revealed a substantial enrichment of downstream genes in glucose metabolism and cell cycle‐related pathways (Figure 5G). These results suggest that HSF1 potentially influences the glucose metabolism process and cell cycle in HCC cells through MYCN, thereby promoting the proliferation of HCC cells.

## 4 DISCUSSION

The mammalian HSF family consists of four members: HSF1, HSF2, HSF3 (found only in Mus musculus), and HSF4.^14^ These factors exhibit unique and overlapping functions, tissue‐specific expression patterns, and undergo various posttranslational modifications.^13^, ^36^, ^37^ Aberrant expression of HSFs is commonly observed in many cancers, influencing growth, metabolism, and HSP expression. In HCC, breast cancer, prostate cancer, lung cancer, esophageal squamous cell carcinoma (ESCC), and colorectal cancer (CRC), HSF2 and HSF4 are frequently dysregulated,^18^, ^38^, ^39^, ^40^, ^41^ while HSF1 is highly expressed across all these cancers.^42^, ^43^ In our study, we analyzed perturbations in the expression of these three HSFs in 22 cancer types and their corresponding adjacent non‐tumor tissues. We found that at least one HSF gene was aberrantly expressed in 15 out of 22 cases. Notably, HSF1, HSF2, and HSF4 were upregulated in HCC, KIRP, and COAD, with HSF1 being upregulated in most tumor types. These findings suggest HSF genes play crucial roles in cancer, and understanding their mechanisms could lead to new treatment strategies.

CNVs are closely associated with tumor development and evolution, affecting the protein levels of genes and promoting tumor growth.^44^, ^45^ Our study demonstrated that HSF1 expression was higher in samples with HSF1 copy number amplification than in samples without amplification in the TCGA–LIHC cohort. HSF1 is likely the most significant member of the HSF family in cancer, exhibiting the most intricate biological functions. Moreover, in multiple groups of HCC samples, HSF1 had a higher expression in HCC compared with adjacent tissues. Patients with high expression of HSF1 had a worse prognosis, their tumors were larger and their AFP levels were higher. These results revealed the importance of HSF1 in HCC from different aspects, and it is necessary to conduct more in‐depth molecular mechanism research on HSF1. Through in vitro and in vivo experiments, we have demonstrated the capability of HSF1 to promote the proliferation of liver cancer cells. Targeting HSF1 in HCC patients may represent a therapeutic strategy for inhibiting the proliferation of HCC cells.

There is a complex mechanism of coordination and interaction among cis‐regulatory elements, transacting factors, RNA polymerases, and histone‐modifying enzymes, which together play a very important role in transcriptional regulation. There are approximately 1600 known TFs in humans^46^ that can bind to promoters and enhancers, recruiting Pol II to initiate transcription. At inactive enhancer regions, chromatin is typically closed and nucleosomes occupy the binding sites of TFs. Therefore, a single TF may not be able to compete effectively with nucleosomes for DNA binding. However, multiple TFs can recognize binding sites within enhancers and collectively evict nucleosomes by “mass action” to achieve collaborative binding.^24^ Meanwhile, histone modifications such as H3K27ac in enhancer regions are also altered due to the co‐occupancy of TFs to facilitate transcription.^27^

In our study, we observed a high degree of overlap between the CUT&Tag peaks of HSF1 and the ChIP‐seq peaks of H3K27ac in the vicinity of the CCAAT motif, suggesting that HSF1 occupancy may modulate H3K27ac at these binding sites. Subsequent experiments confirmed that HSF1 influences the intensity and distribution of H3K27ac in Huh7 cells, leading to alterations in the distribution of SEs as defined by H3K27ac. Transcriptome sequencing further revealed that genes located near these modified SEs exhibited changes in expression. To the best of our knowledge, our study is the first to investigate the regulatory role of HSF1 in SE activity in HCC. We have unveiled a novel function of HSF1, highlighting its ability to function as a chromatin regulator, influencing histone modifications and consequently impacting gene expression. This discovery sheds light on previously unrecognized aspects of HSF1 biology, positioning it as a transcription factor with the potential to modulate the epigenetic landscape in the context of HCC.

Notably, the SE‐related gene most obviously affected by HSF1 was the oncogene MYCN. MYCN has been found to promote tumor growth in a variety of cancers, including prostate cancer, breast cancer, and neuroblastoma.^47^, ^48^, ^49^ In HCC, multiple studies have demonstrated that inhibiting MYCN expression can reduce the proliferation and invasion of liver cancer cells.^50^, ^51^ Moreover, previous studies have shown that MYCN can be used as a biomarker for drug discovery and a therapeutic target for screening chemopreventive agents for HCC recurrence.^52^ Our results confirm that HSF1 promotes HCC cell proliferation through the MYCN pathway and may act through the glucose metabolism pathway or influence the cell cycle. Inhibitor KRIBB11 has shown efficacy in inhibiting HSF1's oncogenic role in HCC.^53^ Furthermore, CDK7 and BET‐Bromodomain inhibitors have exhibited anti‐tumor efficacy by targeting MYCN in various cancers.^54^, ^55^ Histone deacetylase inhibitors (HDACi) have been extensively studied in liver cancer treatment research. Several HDACi, including Valproic acid sodium (VAS),^56^ Panobinostat,^57^ and Vorinostat (SAHA),^58^, ^59^ have been investigated alone or in combination with sorafenib, with ongoing clinical trials.^60^ Given our discovery of HSF1's novel role in epigenetic regulation, combining inhibitors targeting HSF1, MYCN, and HDACs may offer new avenues for exploring therapeutic potentials and clinical value in HCC treatment.

Our study has several limitations that require consideration. Firstly, while we have provided insights into HSF1 protein expression, enriching our dataset with more comprehensive profiling across diverse conditions and stages of cancer progression is necessary to enhance the depth of our findings. Secondly, the absence of transgenic animal models in our experimental design restricted our ability to investigate the complex in vivo interactions and physiological relevance of HSF1 in tumor biology. Lastly, although we identified potential downstream mechanisms involving HSF1, their functional implications have yet to be validated in animal models, necessitating future research efforts to validate these findings and translate them into clinical applications effectively.

**FIGURE 6:**
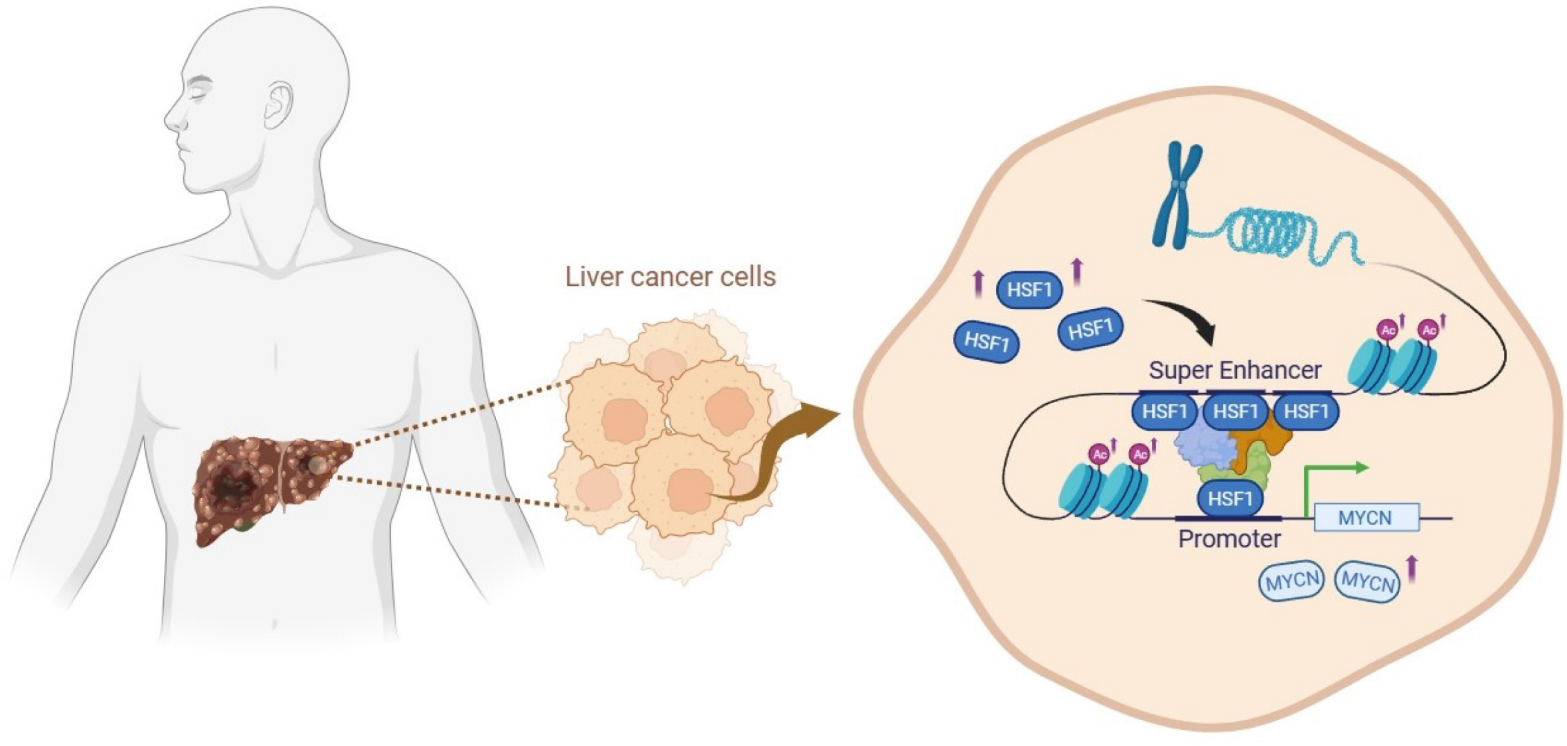
Pattern diagram illustrates the mechanism of the HSF1‐MYCN regulatory axis. HSF1 is upregulated in liver cancer patients. HSF1 can affect the H3K27ac level and the distribution of super enhancers in liver cancer cells, and bind to the super enhancer and promoter region of MYCN to affect its transcription and promote the proliferation of liver cancer cells.

In summary, our findings demonstrate that HSF1 is highly expressed in HCC and modulates the intensity and distribution of H3K27ac, thereby influencing the activity and distribution of SEs. Notably, MYCN emerges as the gene most significantly regulated by HSF1 via SEs. HSF1 regulates MYCN transcription by binding to both its promoter and SE regions, thereby promoting tumor cell growth (Figure 6). This study provides valuable information for the development of future therapeutic strategies for HCC.

## AUTHOR CONTRIBUTIONS

**Yizhe Liu:** Data curation (equal); investigation (equal); validation (equal); visualization (equal); writing – original draft (equal). **Qili Shi:** Data curation (equal); software (equal); visualization (equal); writing – original draft (equal). **Yue Su:** Investigation (equal); validation (equal). **Zhiao Chen:** Conceptualization (equal); data curation (equal). **Xianghuo He:** Conceptualization (equal); project administration (equal).

## FUNDING INFORMATION

This work was supported by grants from the National Natural Science Foundation of China (81930123, 82172937 and 82472641).

## CONFLICT OF INTEREST STATEMENT

The authors declare no conflict of interest.

## ETHICS STATEMENT

This study was approved by the Ethics Committee of the Fudan University Shanghai Cancer Center. Institutional review board approval was obtained and all animal experiments were performed in accordance with institutional guidelines of the Institutional Animal Care and Use Committee of Fudan University Shanghai Cancer Center, Shanghai, China.

## Supporting information


Data S1:

**Table S1:** Primers & siRNAs.

**Table S2:** Antibodies.

## Data Availability

The H3K4me1 and H3K4me3 ChIP‐seq data, as well as the ATAC‐seq data for Huh7 cells, are available under accession number GSE184797. All other data generated and analyzed during this study have been uploaded to the GEO repository under accession number GSE274408.
