# Supplementary material for: Heat shock transcription factor 1 facilitates liver cancer progression by driving super‐enhancer‐mediated transcription of MYCN
Source: Cancer Med. 2024 Sep 9;13(17):e70157. doi: 10.1002/cam4.70157 (PMC11382014; doi:10.1002/cam4.70157)
Supplement: Supplementary file 1 — Data S1: [file CAM4-13-e70157-s002.docx]

**Supplemental Data**

**Heat Shock Transcription Factor 1 Facilitates Liver Cancer Progression by Driving Super-Enhancer-Mediated Transcription of MYCN**

Yizhe Liu^1,4^, Qili Shi^1,4^, Yue Su^1^, Zhiao Chen^1,2,3,*^, Xianghuo He^1,2,3,*^

^1^Fudan University Shanghai Cancer Center and Institutes of Biomedical Sciences; Department of Oncology, Shanghai Medical College, Fudan University, Shanghai 200032, China

^2^Key Laboratory of Breast Cancer in Shanghai, Fudan University Shanghai Cancer Center, Fudan University, Shanghai 200032, China

^3^Shanghai Key Laboratory of Radiation Oncology, Fudan University Shanghai Cancer Center, Fudan University, Shanghai, 200032, China

^4^These authors contributed equally to this work: Yizhe Liu and Qili Shi.

^*^ **Corresponding Authors:**

Xianghuo He, Email: xhhe@fudan.edu.cn or Zhiao Chen, Email: zachen@fudan.edu.cn, Fudan University Shanghai Cancer Center and Institutes of Biomedical Sciences; Shanghai Medical College, Fudan University, 302 Rm., 7# Bldg., 270 Dong An Road, Shanghai 200032, China. Tel: 86-21-34777329; Fax: 86-21-64172585.

**Supplementary Materials and Methods**

**Cell culture**

Huh7 cells were obtained from the Japanese Collection of Research Bioresources (JCRB, Tokyo, Japan). MHCC97L cells were obtained from the Liver Cancer Institute of Zhongshan Hospital (Shanghai, China). HEK-293T cells were obtained from the American Type Culture Collection (ATCC, Manassas, Virginia, USA). All cells were cultured in DMEM (Invitrogen) containing 10% FBS (HyClone) and antibiotics (penicillin and streptomycin, Invitrogen) at a temperature of 37℃. Cells were evaluated monthly for mycoplasma contamination by qRT-PCR analysis using specific primers for mycoplasma detection (Supplementary Table S1). All cells had recently been identified by short tandem repeat analysis.

**Oligonucleotide transfection**

The targeting siRNAs and negative control siRNAs (siNC) were all synthesized by RiboBio (Guangzhou, China). The sequences of siRNAs were shown in Supplementary Table S1. Approximately 3×105 cells were seeded into the six-well plate and grew for one day. At 30% of confluence, 5μL siRNA (20μM) were transfected into cells using Lipofectamine RNAiMAX (Invitrogen). After 48 h, cells were harvested for further experiments.

**Western Blot**

Proteins were separated by SDS-PAGE and then transferred to nitrocellulose mem-brane (Bio-Rad, Hercules, CA, USA). The nitrocellulose membranes were blocked in 5% non-fat milk at room temperature for 1 hour and incubated with primary antibodies at 4℃ overnight. The membranes were incubated with secondary antibodies at room temperature for 1 hour the next day. The antigen-antibody complex was detected by a LumiBest ECL Reagent Solution Kit (Share-Bio, Shanghai, China). Antibodies used in this study were listed in Supplementary Table S2.

**CRISPR–Cas9-mediated deletion of enhancers**

CRISPR-ERA (http://crispr-era.stanford.edu/) was used to design the sgRNAs. The sgRNAs used to delete enhancers were annealed with 10× annealing buffer [1 M Tris-HCl (pH 8.0), 5 M NaCl, and 500 mM EDTA (pH 8.0)] at 95°C for 3 minutes, and the mixture was then allowed to cool naturally to room temperature. Annealed double-stranded DNA was inserted into the Lenti-gRNA-puro vector (Addgene), which was digested with BsmBI (NEB). At 30-40% confluence (60% confluence for MHCC97L-Cas9), Huh7-Cas9 cells in 6-well plates were infected with viruses packaged with purified recombinant plasmids containing the sgRNAs. After 48 hours, puromycin was added to the cells, which were collected to assess the knockdown efficiency and gene expression. The primer sequences are listed in Supplementary Table S1.

**Construction of the enCRISPRi cell line**

First, lentiviruses were packaged with pHR-SFFV-KRAB-dCas9-P2A-mCherry (Addgene) and Lenti_MCP-LSD1_Hygro (Addgene). After infecting Huh7 and MHCC97L cells with both viruses, we selected hygromycin-resistant and mCherry-positive cells and eventually obtained dCas9-KRAB-LSD1 cells. Afterward, we determined the locations of the enhancers and obtained the DNA sequences from the corresponding ATAC-seq peaks. We then designed sgRNAs targeting the enhancer from these DNA sequences using CRISPOR (http://crispor.tefor.net/crispor.py). Annealed double-stranded DNA was inserted into Lenti-sgRNA(MS2)-ZsGreen1 (Addgene), which was digested with BsmBI (NEB). Then, viruses packaged with the purified recombinant plasmid were used to infect dCas9-KRAB-LSD1 cells in 100 mm dishes. ZsGreen-positive cells were selected by flow cytometry, and the variation in the mRNA level of MYCN was obtained with qRT-PCR. The primer sequences are listed in Supplementary Table S1.

**Supplementary Figures**


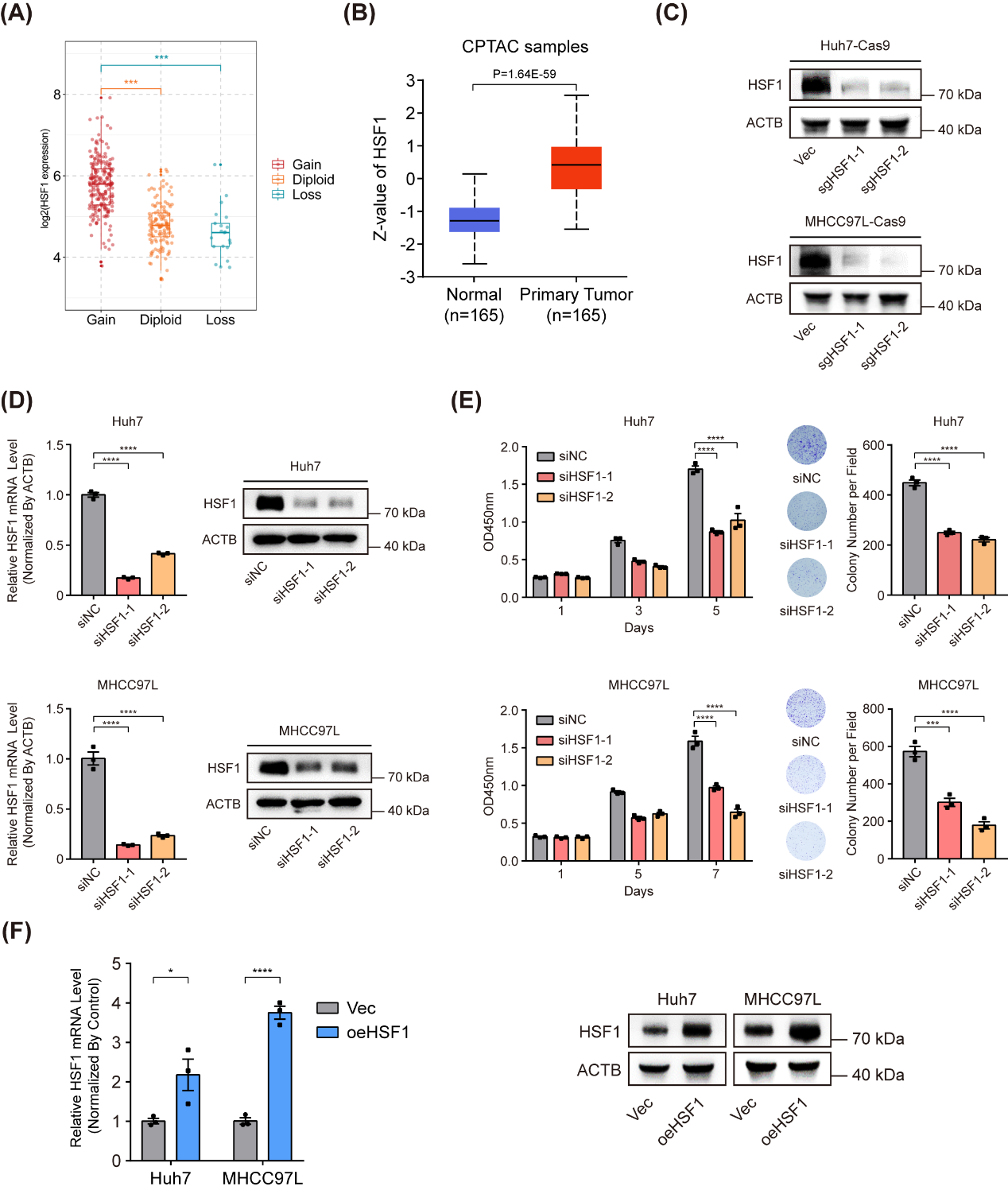


**Supplement Figure S1**

(A) The expression level of HSF1 in different copy number amplification groups in the TCGA-LIHC cohort. (B) Protein expression of HSF1 in primary tumor tissue and normal tissue in CPTAC samples from UALCAN database. Z-values represent standard deviations from the median across samples for the given cancer type. Log2 Spectral count ratio values from CPTAC were first normalized within each sample profile, then normalized across samples. (C) HSF1 protein expression levels were determined by Western blot analysis in two liver cancer cell lines after HSF1 knockout. (D) The qRT-PCR and Western blot analysis of the HSF1 knockdown efficiency after transfection of Huh7 and MHCC97L cells with two independent HSF1 siRNAs. (E) CCK-8 assays and colony formation assays of the two liver cancer cell lines after HSF1 knockdown (n = 3). (F) The overexpression efficiency of HSF1 levels in Huh7 and MHCC97L cells was determined by qPCR and Western blot (n = 3). The values are expressed as the means ± SEMs (A, D-F). *, P < 0.05; ***, P < 0.001; ****, P<0.0001 by one-way ANOVA or two-tailed Student’s t test.


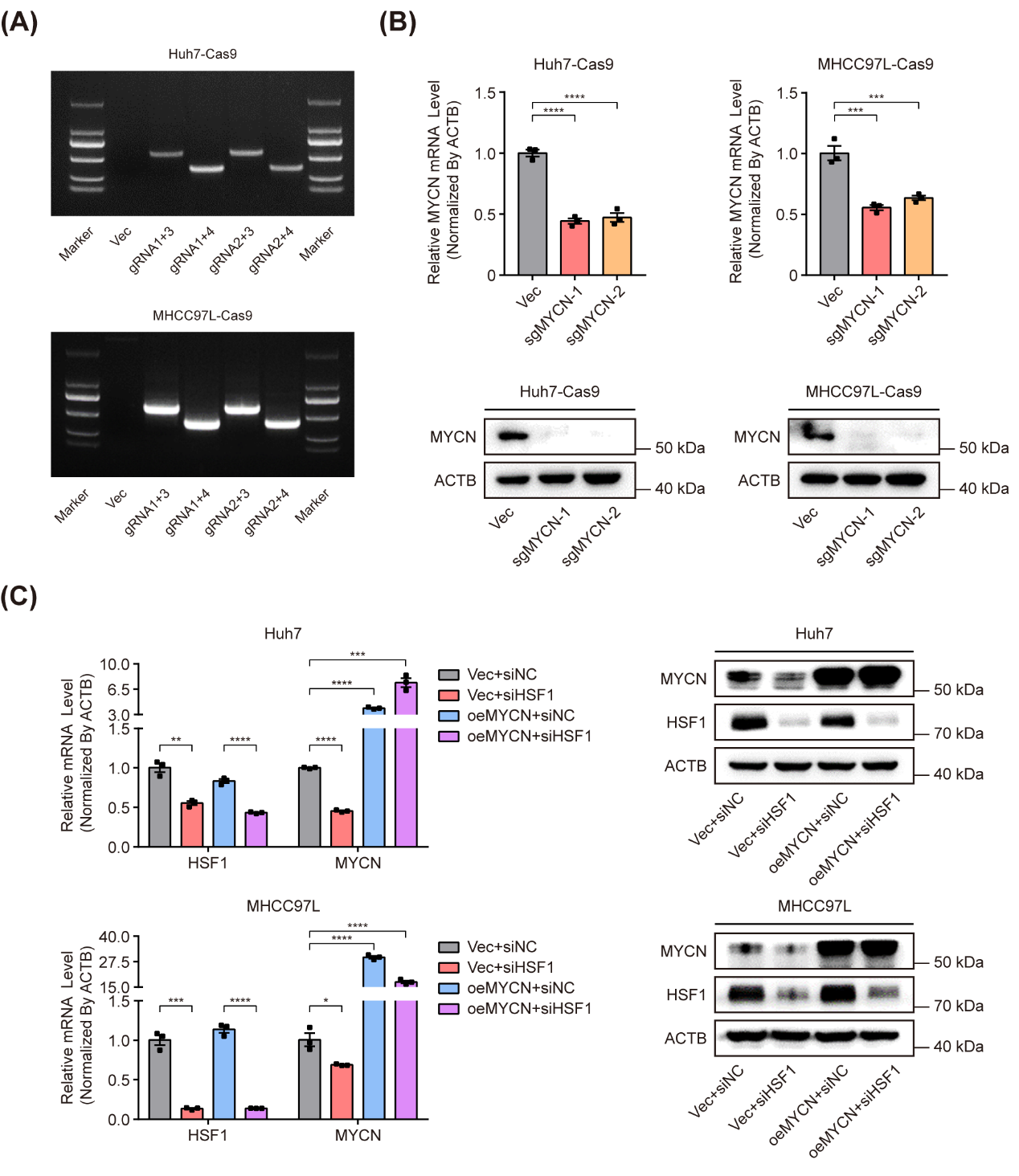


**Supplement Figure S2**

(A) Validation of the knockout efficiency in Huh7-Cas9 and MHCC97L-Cas9 cells transfected with four combinations of sgRNAs by agarose gel electrophoresis of extracted genomic DNA. (B) qRT-PCR and Western Blot analysis of MYCN knockout efficiency by CRISPR-Cas9 gene editing in Huh7-Cas9 and MHCC97L-Cas9 cells with two independent MYCN sgRNAs (n = 3). (C) The mRNA and protein expression levels of HSF1 and MYCN in Huh7 and MHCC97L cells from four different treatment groups (n = 3). The values are expressed as the means ± SEMs (B, C). *, P < 0.05; **, P < 0.01; ***, P < 0.001; ****, P<0.0001 by one-way ANOVA or two-tailed Student’s t test.
